# Analysis of a Repetitive Language Coding System: Comparisons between Fragile X Syndrome, Autism, and Down Syndrome

**DOI:** 10.3390/brainsci12050575

**Published:** 2022-04-29

**Authors:** Anne Hoffmann, Angela John Thurman, Audra Sterling, Sara T. Kover, Lizabeth Finestack, Elizabeth Berry-Kravis, Jamie O. Edgin, Andrea Drayton, Eric Fombonne, Leonard Abbeduto

**Affiliations:** 1 Departments of Communication Disorders and Sciences and Pediatrics, Rush University Medical Center, Chicago, IL 60612, USA; 2 MIND Institute, School of Medicine, University of California Davis Health, Sacramento, CA 95817, USA; ajthurman@ucdavis.edu (A.J.T.); andrayton@ucdavis.edu (A.D.); ljabbeduto@ucdavis.edu (L.A.); 3 Department of Psychiatry and Behavioral Sciences, University of California Davis Health, Sacramento, CA 95817, USA; 4 Waisman Center, Department of Communication Sciences and Disorders, University of Wisconsin-Madison, Madison, WI 53706, USA; audra.sterling@wisc.edu; 5 Department of Speech and Hearing Sciences, University of Washington, Seattle, WA 98195, USA; skover@uw.edu; 6 Department of Speech-Language-Hearing Sciences, University of Minnesota, Minneapolis, MN 55455, USA; feinstac@umn.edu; 7 Departments of Pediatrics, Neurological Sciences, Anatomy and Cell Biology, Rush University Medical Center, Chicago, IL 60612, USA; elizabeth_m_berry-kravis@rush.edu; 8 Department of Psychology and Sonoran UCEDD, College of Science, The University of Arizona, Tucson, AZ 85721, USA; jedgin@email.arizona.edu; 9 Departments of Psychiatry and Pediatrics, School of Medicine, Oregon Health and Science University, Portland, OR 97239, USA; fombonne@ohsu.edu

**Keywords:** fragile X syndrome, autism, down syndrome, expressive language sampling, pragmatic language, repetitive language

## Abstract

Expressive language sampling (ELS) is a frequently used tool for language analysis, as it can be used across widely ranging cognitive and language abilities. ELS can also evaluate pragmatic language, including excessive self-repetition, which is challenging to assess with traditional standardized assessments. This study explored how a well-established ELS protocol can assess three types of linguistic self-repetition in three neurodevelopmental disabilities: fragile X syndrome (FXS), autism spectrum disorder (ASD), and Down syndrome (DS). We examined its ability to differentiate between these disorders, the relationships between repetitive language and other participant characteristics, and initial construct validity. We found that the groups with FXS and ASD differed significantly on each of the three repetitive language measure, and that the group with DS differed from either ASD or FXS on two. Cognitive ability was significantly related to phrase repetition in the group with ASD. When the groups were combined, there was evidence of convergent and divergent validity. This study extends previous research on ELS and supports its use as a means to characterize pragmatic language. It also provides information about the relationships between repetitive language and other phenotypic characteristics.

## 1. Introduction

The study of language development and usage has uncovered similarities and differences between various neurodevelopmental disorders [1,2,3,4]. As language touches on multiple domains (e.g., cognition, socialization, activities of daily living), understanding its phenotypic expression can inform both theoretical understanding of neurodiversity impacts on development as well as clinical practice. Expressive language sampling (ELS) has long been believed to represent a more functional form of assessment as compared to traditional standardized and norm-referenced assessments [5,6]; ELS also allows for comparison of language use in naturalistic settings between diagnostic groups. Using ELS, the form (syntax, morphology), content (vocabulary), and use (pragmatics) of language have all been analyzed through various coding methods [7,8,9,10,11]. This study focused on one aspect of pragmatic language thought to distinguish diagnostic groups: linguistic self-repetition (hereafter, referred to simply as “repetitive language”). 

### 1.1. Expressive Language Sampling

ELS refers to any method that allows for the collection of a naturalistic sample of verbal speech and is a frequently used tool in both research and clinical practice as it allows for characterization of both developmental change and impairments [7]. However, there have been challenges in how to best standardize elicitation procedures to remove potential confounding factors and maintain adequate consistency in administration across participants and occasions of measurement to ensure valid and reliable assessment. The ELS procedures outlined by Abbeduto, Thurman, and colleagues have been shown to meet these criteria and to be feasible and psychometrically sound for individuals with various neurodevelopmental disorders such as fragile X syndrome (FXS), autism spectrum disorder (ASD), and Down syndrome (DS) [11,12]. 

A key strength of ELS is that it can be used with negligible change in procedures across a wide range of age, intellectual ability, and language ability. This is vital as tools able to measure language skills across the range of verbal ability without significant floor or ceiling effects are extremely limited [13]. This gap in psychometrically sound assessment options has been identified as one of the greatest factors in the failure of multiple clinical trials targeting various developmental disabilities [5,14]. Also, standardized ELS procedures can be used to analyze pragmatic language, which is frequently noted as a relative weakness in both FXS and ASD and a possible strength in DS [8,15,16]. Pragmatic language refers to the functional use of language, essentially knowing when to speak, what to say, and how to say it [17]. Because pragmatic language relies on in-context communication, the decontextualized nature of most traditional standardized language assessments eliminates the possibility of obtaining an objective generalizable measure of pragmatic language skills. More naturalistic contexts for assessing pragmatic language have been recommended for research and clinical practice, making ELS an ideal candidate [18,19,20,21,22].

Previous studies that have examined pragmatic language analysis using ELS have found that it distinguishes between various syndromes [8,10,23,24,25]; however, some elements of pragmatic language have proven to be more challenging to characterize, including verbal self-repetition—also termed perseveration—which refers to the excessive self-repetition of a spoken word, phrase, sentence, or topic. Verbal self-repetition (hereafter, simply “repetitive language”), has been noted as prevalent in both ASD and FXS [26,27,28,29]. Previous studies have differed in how repetitive language is defined, coded, and in how the language samples are elicited. The present study was designed to examine how a standardized coding schema using a standardized and psychometrically validated ELS protocol could potentially differentiate between groups with neurodevelopmental disorders in terms of repetitive language. Specifically, we focused on repetitive language in FXS, ASD, and DS.

### 1.2. FXS

FXS is the most common inherited cause of intellectual disability, occurring in approximately 1/2500–1/5000 individuals [30,31,32]. This neurodevelopmental disorder is the result of a trinucleotide expansion in the *FMR1* gene that results in the reduction or absence of the fragile X messenger ribonucleoprotein (FMRP), a critical factor in brain development and synaptic plasticity [33]. Because of its X-linked nature, females with FXS are often less affected than males, although both sexes present with a wide range of impairments in functioning [34,35]. FXS also shares many characteristics with ASD with up to 60% of males with FXS meeting criteria for an ASD diagnosis and up to 90% showing autistic symptomatology [36,37,38]. 

As would be expected given the high rate of ASD diagnoses, the FXS phenotype includes specific weaknesses in pragmatic language—a hallmark of ASD. Notably, repetitive language has been reported as a defining characteristic of FXS [9,10,28,39,40,41]. There is some evidence that sex differences in repetitive language exist that cannot be explained by overall level of functioning. Murphy and Abbeduto [42] (2007) examined repetitive language in a group of 16 boys and 8 girls with FXS and found that the boys produced significantly higher amounts of conversational devices. This was not explained by level of intellectual disability or linguistic ability but seemed to be a specific difference between genders. However, Martin and co-authors [10] compared boys and girls with FXS and FXS+ASD and did not find significant differences based on gender for phrase or topic repetition, so it may be a matter of form of repetition. 

The question of whether repetitive language varies within FXS based on ASD symptomatology has also had mixed responses. Roberts et al. [40] found that individuals with FXS only (FXS-O) demonstrated the same levels of repetitive language coded turn by turn as those with FXS and co-occurring ASD (FXS+ASD), however this study collapsed all forms of repetition into one category. Two studies by Martin and colleagues [9,10] found increased levels of topic repetition in boys with FXS+ASD as compared to those with FXS-O when coding was done turn by turn. Klusek et al. [8] used a summary score based on ratings rather than frequency counts of key behaviors observed in a video-taped semi-structured conversation to measure repetitive language, this work did not find differences between boys with FXS-O and boys with FXS+ASD. These varying findings may reflect the differences in coding procedures of those studies (i.e., turn-by-turn hand coding vs. a summary code) or participant characteristics (e.g., age or IQ).

### 1.3. ASD

ASD is a behaviorally diagnosed neurodevelopmental disorder with an estimated prevalence of 1 in 44 [43]. The diagnosis requires significant differences in social-communication and restricted/repetitive behaviors, which impact the ability to participate in daily activities [44]. Language development varies widely in this population, with some individuals exhibiting language that falls within or above age expectations, whereas other individuals have very limited verbal communication [45]. Language ability is often correlated with cognition; however, there are also instances in which language outpaces cognition and vice versa [46]. Heterogeneity in clinical presentation also stems from sex, as females with ASD have been noted to present with differences in manifestation of autistic symptomatology [47].

Pragmatic language, which is a subset of social communication skills, is impaired in ASD by definition, with repetitive language being one characteristic noted [48]. Comparisons between ASD and FXS using language sampling have yielded mixed results. Sudhalter et al. [28] reported that individuals with FXS had higher rates of perseveration than that seen in ASD, but no distinction was made as to ASD diagnosis for the participants with FXS. Martin and colleagues [9,10] Another study examining boys and adolescents with either FXS+ASD or ASD found that the males with FXS+ASD were more likely to use repetitive language [49]. Other studies have found similar rates of repetitive language in FXS+ASD as compared to ASD [9,10,50]. Differences in operational definitions of repetition and participants characteristics may account for inter-study variability in findings. It is also important to acknowledge the fact that self-repetition and echolalia differ in that the former is defined by the individual repeating their own utterances excessively, and not necessarily imitating another person.

### 1.4. DS

DS is the most common known genetic cause of intellectual disability, occurring in 1 of 700–800 live births [51]. Although expressive language, particularly expressive syntax, is a specific area of weakness, social skills, including pragmatic language, are often considered a relative strength in this population [15,52,53,54]. Previous analyses of language samples have revealed that boys with DS use less repetitive language than boys with FXS or ASD [9,28,40,55]. Further, Klusek et al. [8] did not find differences in repetitive language between boys with DS and boys with TD matched on mental age; again, perhaps suggesting that repetitive language in DS may be less common than what is seen in other groups such as FXS and ASD. That said, there has historically been a dearth of research considering the use of repetitive language in individuals with DS. However, there is a growing body of research indicating that the prevalence of ASD in individuals with DS is higher than that observed for the general population [56], which raises the possibility of higher rates of repetitive language in this population as well. 

### 1.5. Current Study

It is likely that the differences in findings across studies are due to the different methods used to collect the samples and assess verbal self-repetition, e.g., standardization of the elicitation protocol, turn-by-turn vs. summary coding, etc. Indeed, the sampling context of ELS (e.g., narration vs. conversation) has been shown to affect the type and amount of repetition elicited [24,57]. Even within studies, there have been uncontrolled differences in the sampling context across participants [9]. The ways in which repetitive language is operationalized may also affect results [57] and jeopardize between-studies comparisons. The present study was designed to provide an increased understanding of diagnostic group differences and to establish the initial construct validity of ELS-derived repetition measures. This study used previously validated and standardized ELS procedures in conjunction with a new coding schema based on the parameters of repetition detailed in Murphy & Abbeduto [4], which distinguished between repetition of phrases, topics, and conversational devices, as these repetition types may vary with diagnostic group. We addressed three questions:

1. Does repetitive language as measured using ELS conversation distinguish between FXS, ASD, and DS?

Hypothesis: Significant between-group differences will be found on measures of repetitive language coded from the ELS conversation for FXS, ASD, and DS, with the highest rates for individuals with FXS given the cooccurrence of intellectual disability and ASD. 

2. Does repetitive language as measured using ELS conversation correlate with participant characteristics including those reflecting various dimensions of ability (e.g., nonverbal cognitive ability) or challenge (e.g., autistic symptomatology)?

Hypothesis: Significant correlations will be found between repetitive language measures coded from the ELS conversation and participant characteristics including those reflecting various dimensions of ability or challenge.

3. Does repetitive language as measured using ELS conversation demonstrate initial construct validity?

Hypothesis: Significant correlations will be found between repetitive language coded from the ELS conversation and informant report measures of repetitive language but not with a conceptually unrelated informant report measure. 

## 2. Materials and Methods

### 2.1. Participants

Data analyzed for this study were derived from a larger sample recruited to examine the psychometric properties of standardized ELS procedures (e.g., Abbeduto et al. [12]) using a multisite design of seven different universities (ASD: *n* = 79, DS: *n* = 107, FXS: *n* = 106). The participants included in the present project consisted of all participant samples coded at the time of study analysis (ASD: *n* = 30, DS: *n* = 34, FXS: *n* = 29). The study was approved by the Institutional Review Boards of all participating universities and written informed consent from the parent/guardian of all participants and participant assent were obtained prior to beginning study procedures. 

Inclusionary criteria across the three diagnostic groups included a chronological age (CA) between 6 and 23 years at first testing and caregiver report of (1) speech as the primary mode of communication, (2) production of occasional three-word or longer utterances, (3) English as the primary language spoken in the home, (4) no more than a mild hearing loss, and (5) no serious (uncorrected) visual impairments that would preclude successful performance on the testing battery. In addition, for participants with DS or FXS, (1) parents provided genetic reports confirming the diagnosis and (2) reported or provided records documenting an IQ within the ID range (IQ ≤ 70), with the IQ criterion subsequently confirmed through direct testing at the initial visit. For participants with ASD, caregivers provided a report confirming a clinical diagnosis of ASD by an appropriate community provider, with the diagnosis subsequently confirmed through direct testing (i.e., the ADOS-2) at the initial visit. IQ was not constrained for participants with ASD. Two participants in the ASD group and one in the FXS group did not meet criteria at the initial visit and were excluded from analysis. Of the participants with FXS, 21 of the 28 with ADOS-2 results met criteria for ASD, in the group with DS, 12 of the 29 with ADOS-2 results met criteria for ASD. Testing was performed at each location by examiners highly experienced with developmental disabilities and assessment in these populations and trained to fidelity on ELS procedures (see Abbeduto et al. [12] for details). A between-groups Kruskal Wallis test was run for each of the participant characteristics, with significant differences for each characteristic except CA. Participant characteristics are shown in Table 1.

### 2.2. Measures

#### 2.2.1. Expressive Language Samples

Language samples were collected using the conversation procedure of the ELS protocol (see Abbeduto et al. [12] for a full description of the procedures for administration, training of examiners, and determining fidelity of administration). In this assessment, the examiner introduces a series of pre-determined topics after beginning the conversation with an idiosyncratic topic relating to the specific interests of the participant according to parent report. The examiner follows a script for introducing and following up on the topics and for minimizing their talk while encouraging the participant to talk, thereby achieving standardization across examiners, participants, and occasions of measurement. The examiners are instructed to continue the conversation task for 12 min (to facilitate acquiring a 10-min sample of participant speech) and all examiners administering the procedure met fidelity standards as outlined in Abbeduto et al. [12]. Transcriptions of the first 10 min of each sample were created using Systematic Analysis of Language Transcripts (SALT; [57]). See Abbeduto et al. [12] for details of the transcription process. Utterances were segmented into C-units (Communication-units), which can range from a single word to an independent clause with its modifiers including subordinate clauses. 

The complete and intelligible utterances identified in the transcripts (ASD: M = 90.63, SD = 41.46; DS: M = 91.65, SD = 30.02; FXS: M = 108.76, SD = 38.99) were then coded for the following types of repetitive language: Phrase Repetition, Topic Repetition, and Conversational Device Repetition. See Table 2 for details and examples of each repetition type. Again, it is important to recognize that interest was in self-repetition rather than in repetition or imitation of others. All transcripts were initially drafted by a primary transcriber, reviewed and edited by a secondary transcriber, and then finalized by the primary transcriber, creating the transcripts used in these analyses. Coders then went through each transcription to identify the repetition types described below. These measures were coded from absolute frequency of the specific self-repetition type. Intraclass Correlation Coefficient (ICC) estimates and their 95% confidence intervals were computed, for 10 samples, based on a mean-rating (k = 2), absolute-agreement, 2-way mixed effects model. Results indicate excellent reliability for the total number of Phrase Repetitions (ICC = 0.999; 95% confidence interval, 0.995–1.00), Conversational Devices (ICC = 0.994; 95% confidence interval, 0.963–0.999), and Topic Repetitions (ICC = 0.979; 95% confidence interval, 0.728–0.996). 

*Phrase Repetition* was characterized as phrases (including single-word C-units), dependent clauses, and/or entire C-units that the participant said more than once in immediate succession. The repetition had to maintain essentially the same structure and no new substantive semantic content could be added relative to the participant’s previous C-unit, as in “They must have fainted too. They must have fainted” [42]. A minor alteration (a difference of one morpheme that does not change the meaning of the utterance) of the phrase was allowed. Only self-repetition was coded–echolalia, or repetition of another’s speech–was excluded. For Phrase Repetition, the following variables were computed: the total number of C-units containing a phrase repetition (Phrase Total), the average number of repetitions of a specific phrase (Phrase Average), and the maximum number of repetitions of a specific phrase (Phrase Max). 

*Topic Repetition* was characterized by continued talking about a topic even when the conversation had moved on to other things or repetition of topic details that had already been established. In coding topic repetition, all utterances that pertained to a topic/theme/idea that the child talked about in at least three C-units were identified. Topic Repetition was then coded when a C-unit identified as pertaining to a topic met one of three criteria: (1) it offered no new information relative to the child’s or examiner’s prior C-unit; (2) it was non-contingent to the previous statement by the examiner; or (3) it interrupted the examiner. The total number of C-units meeting criteria for Topic Repetition was calculated for each participant. 

*Conversational Device (CD) Repetitions* are rote sayings and phrases that help direct the interaction but do not add content to the topic being discussed, as in “right on” [42]. These were considered repetitive if a participant uttered the same conversational device three or more times within the interaction between examiner and participant (i.e., the entire transcribed conversation). For this type of repetition, the total number of C-units containing a conversational device repetition (CD Total) and the maximum number of C-unit repetitions using a specific conversational device (CD Max) were analyzed. 

#### 2.2.2. Stanford-Binet, Fifth Edition

The Stanford-Binet-Fifth Edition (SB5; [58]) was used to measure participants’ cognitive abilities. This widely-used measure of intelligence and cognitive ability uses 10 subtests to measure five weighted factors. These factors include knowledge, quantitative reasoning, visuo-spatial processing, working memory, and fluid reasoning and are assessed using verbal and nonverbal subtests. Nonverbal, verbal, and full-scale intelligence quotients are derived from subtest scores. Nonverbal intelligence quotients (NVIQ) were used in analyses to minimize the effect of shared method variance with the ELS procedures. Because of the floor effects on this assessment, z-derived scores were calculated, which allow for greater precision of IQ measurement [59,60]. Within the participants, six individuals with DS and two individuals with FXS were missing SB5 results. 

#### 2.2.3. Autism Diagnostic Observation Schedule-Second Edition

The Autism Diagnostic Observation Schedule-Second Edition (ADOS-2, [61]) provided a measure of autistic symptomatology. This semi-structured observation measure uses a series of activities presented by a research-reliable examiner to elicit a sample of social affect (SA) and restricted repetitive behaviors (RRB). Participants were assessed using the module appropriate to their age and communication ability, as specified by the examiner’s manual. The total scores for the RRB and SA scales were used as a metric of autistic symptomatology in those respective areas with higher scores indicative of increased symptom presence. Within the participants, five individuals with DS and one individual with FXS were missing ADOS-2 results.

#### 2.2.4. Vineland Adaptive Behavior Scales-Second Edition 

The subscale Expressive Communication from the Vineland Adaptive Behavior Scales-Second Edition (VABS-II; [60]) was used as a measure of expressive language ability. The VABS-II is an informant-report measure that yields an adaptive behavior composite score as well as domain scores (v-scores, with a mean of 15 and standard deviation of 3). This measure has been shown to be valid and reliable and has been used extensively in clinical care and research to measure the development and functioning of individuals with and without disabilities [62,63,64]. In this study, the informant was either a parent or a legally authorized representative. V-scores were used in analysis as examination of the data indicated that only two participants received the lowest possible score on this subtest. The v-scores permit a better understanding of language variation among participants. Note that the data for this study were collected before the Vineland-3 was available. 

#### 2.2.5. Aberrant Behavior Checklist 

Two items from the Aberrant Behavior Checklist (ABC, [65]) subscale of Inappropriate Speech were combined to measure caregiver perception of repetitive speech. Items 22 (*Repetitive speech*) and 46 (*Repeats a word or phrase over and over*) were added together to create one measure of repetition. Caregivers rate each of these items on a scale of 0 to 3 with higher scores being indicative more problematic behaviors (e.g., a rating of “0” corresponded to something “not a problem”, a rating of “3” corresponds to a “severe problem”). The combined raw score from the two items was used in the analyses to assess convergent validity. Discriminant validity was assessed using the total raw score for Irritability, a subscale measuring behaviors such as verbal outbursts, physical aggression, and tantrums. 

### 2.3. Data Analysis

Analyses were completed using either SAS/STAT (median regression analyses) or SPSS version 26 (correlations). As the data violated the assumption of normality, analyses appropriate for non-parametric distributions were used throughout. In order to assess potential group differences in the occurrence of the different types of repetitive language, median regressions were used to determine if significant difference remained between diagnosis groups after controlling for total number of C-units. We controlled for the total number of C-units as fewer total utterances might cause the appearance of less repetition when the reverse was true in a proportional sense. For example, an individual who produced three C-units, but each of them contained the same repeated phrase would receive the same frequency score as an individual who produced 30 C-units that contained three repetitions of the same phrase. Planned comparisons were conducted following each median regression even if the overall model was nonsignificant given our hypotheses and to guard against Type 2 error.

To assess the relationship between repetition and other participant characteristics, Spearman’s rho correlations were calculated between repetition scores and restricted and repetitive behavior as measured by the ADOS-2 (ADOS-RRB), social affect as measured by the ADOS-2 (ADOS-SA), NVIQ, expressive language as measured by the VABS-II, and CA with C-units once again used as covariate. These two areas of the ADOS-2 were chosen as they are representative of the core areas of autistic symptomatology. In order to determine if patterns were reflective of intellectual disability and not phenotypic characteristics, additional Spearman-rho correlations were also calculated for the ASD group with NVIQ at and above 70 (ASD-O, *n* = 10) and the ASD group with NVIQ below 70 (ASD+ID, *n* = 20). 

The relationship between repetitive language coded from the ELS and other measures of repetition was also investigated using Spearman’s rho correlations between repetition measures and the combined items from the ABC Inappropriate Speech subscale. As this question focused on the construct validity and not necessarily group specific characteristics, the analyses were performed both within the different groups and with the FXS, ASD, and DS groups combined into one group and results were again corrected for the number of C-units. Discriminant validity was assessed using Spearman’s rho correlations corrected for number of C-units and a subscale of the ABC not associated with repetitive language; namely, Irritability. 

In each of these analyses, we used Benjamini and Hochberg’s false discovery rate (FDR) [66] to correct for multiple comparisons. This procedure maintains a familywise alpha rate of *p <* 0.05. In using the FDR, we defined the family in the following way: (1) the median regressions were considered one family, as were the pairwise testing following each median regression that indicated a significant difference between groups; (2) For correlations, each participant group and the analyses addressing Questions 2 and 3, respectively, were each considered to be one family (e.g., for the group with DS, participant characteristics and correlations with ELS Phrase Repetition measures were considered one family, as were ABC items and ELS repetition variables).

Dividing the FXS group based on ASD diagnosis was considered; however, analyses revealed that there were no significant differences in any of the repetition measures when FXS-O and FXS+ASD were compared. Thus, the FXS group was kept as a whole throughout the analyses described below. 

## 3. Results

### 3.1. Does Repetititive Language as Measured Using ELS Conversation Distinguish between FXS, ASD, and DS?

#### 3.1.1. Phrase Repetition

The median regressions revealed significant differences between groups for Phrase Total, Phrase Average, and Phrase Max (see Table 3). Pairwise comparisons following the median regressions demonstrated that the group with FXS was significantly higher than the group with ASD for Phrase Total (*p* = 0.0002)), Phrase Average (*p* < 0.0001) and Phrase Max (*p* < 0.0001). The group with DS was significantly lower than the group with FXS for Phrase Total (*p* = 0.04), Phrase Average (*p* = 0.004), and Phrase Max (*p =* 0.004) and significantly higher than the group with ASD for CD Max (*p* < 0.0001). All differences remained significant except for that of Phrase Total between the groups with FXS and DS after correction for FDR.

#### 3.1.2. Topic Repetition

Median regressions demonstrated a significant difference between groups for Topic Repetition (see Table 3), however this did not remain significant after correction for FDR. Per our analysis plan, we completed pairwise comparisons that demonstrated that the group with FXS was significantly higher than the group with ASD for Topic Repetition (*p* = 0.01) this remained significant after correction for FDR.

#### 3.1.3. Conversational Devices

No significant difference was found between groups for CD Total, nor did pairwise comparisons find significant differences (see Table 3). The median regression did reveal a significant difference between groups for CD Max (see Table 3). Follow-up pairwise comparisons demonstrated that the group with ASD was significantly higher than the group with FXS (*p* < 0.0001) and the group with DS (*p* < 0.0001) for CD Max and this remained significant after correction for FDR. 

### 3.2. Does Repetition as Measured Using ELS Conversation Correlate with Participant Characteristics including Those Reflecting Various Dimensions of Impairment?

#### 3.2.1. Correlations between Repetitive Language Measures and Participant Characteristics

Correlations between Phrase Repetition measures and ADOS-RRB, ADOS-SA, CA, NVIQ, and expressive language were calculated. In the group with ASD, significant correlations were found between each Phrase Repetition measure and NVIQ and these remained significant after FDR corrections. No other correlations remained significant after FDR correction. 

Correlations between Topic Repetition and ADOS-RRB, ADOS-SA, CA, NVIQ, and expressive language were calculated. There were no significant correlations between Topic Repetition and any participant characteristic measure for any of the groups after correction for FDR. 

Finally, correlations between CD repetition measures and ADOS-RRB, ADOS-SA, CA, NVIQ, and expressive language were calculated. After FDR corrections, there were no significant correlations between either CD Repetition measure and the participant characteristic measures for any of the groups after the FDR correction. All correlations are shown in Table 4 and Table 5.

#### 3.2.2. Correlations after Dividing the ASD Group Based on NVIQ

To assess the possibility of different patterns within the ASD group given the wider IQ range and the significant correlations observed between NVIQ and the Phrase Repetition measures, participants were then separated into ASD-O and ASD+ID. After this, there was no longer a significant correlation between repetition measures and any of the participant characteristics including NVIQ in either subgroup, although the sample size was smaller secondary to the group separation. When examining the correlation coefficients, the ASD+ID group has larger correlation coefficients than both the ASD-O group and ASD combined group for Phrase Repetition measures and NVIQ, ADOS-RRB, ADOS-SA, CA, and expressive language. Correlations are shown in Table 6. 

### 3.3. Does Repetition as Measured Using ELS Conversation Demonstrate Initial Construct Validity?

#### 3.3.1. Convergent Validity

Correlations were calculated between all repetitive language measures and the ABC summed score for Inappropriate Speech. There were no significant correlations between the ABC combined score and Phrase Repetition measures for any of the individual diagnostic groups. When groups were combined, however, the ABC combined score was significantly correlated with Phrase Total (*ρ* = 0.256, *p* = 0.013), Phrase Mean (*ρ* = 0.294, *p* = 0.004), and Phrase Max (*ρ* = 0.278, *p* = 0.007), and these correlations remained significant after FDR correction.

No significant correlations were found between the ABC combined score and Topic Repetition for either the individuals or combined groups. 

Significant correlations were found between the ABC combined score and CD Total in the group with ASD (*ρ =* 0.370, *p* = 0.048). When the groups were combined, the ABC combined score was significantly correlated with CD total (*ρ =* 0.241, *p* = 0.02) and CD Max (*ρ* = 0.223, *p* = 0.032), but no correlations remained significant after FDR correction. Correlations are shown in Table 7. 

#### 3.3.2. Discriminant Validity

Correlations were calculated between the repetitive language measures and the ABC subscale for Irritability. There were no significant correlations between the Irritability and Phrase Repetition measures for any of the individual diagnostic groups or the combined group. 

No significant correlations were found between the ABC Irritability subscale and Topic Repetition for either the individual or combined groups. 

A significant correlation was found between the Irritability subscale and CD Total in the group with DS (*ρ* = 0.399, *p* = 0.046). This did not remain significant after correction for FDR. When the groups were combined, there were no longer any significant correlations. Correlations are shown in Table 8.

## 4. Discussion

This study adds to the overall understanding of how pragmatic language, and more specifically repetitive language, can differ between neurodevelopmental disorders, and how we can effectively assess this behavior. Our first question was whether repetitive language based on measures derived from the ELS conversation samples [12] could distinguish between individuals with FXS, DS, or ASD. Our hypothesis was supported in that clear differences between groups emerged, most consistently in Phrase Repetition. The difference in repetitive language between groups was most consistent for ASD versus FXS. Each area of repetition (e.g., Phrase Repetition, Topic Perseveration, Conversational Device Repetition) was significantly different between the two groups, with the group with FXS having higher rates for the total amount of phrase repetition as well as the greatest number of repetitions of a specific phrase and the greatest number of C-units containing topic perseverations. The participants with ASD produced the most repetitions of a specific conversational device, being significantly different from both the group with FXS and the group with DS. Given the highly overlapping nature of many behavioral characteristics in FXS and ASD, our findings suggest that repetitive language is a valuable measure when attempting to distinguish the two phenotypes. At the same time, the findings suggest that higher rates of repetitive language are not simply due to higher rates of ID. This is implied by the fact the only significant difference in Conversational Devices actually favored the participants with FXS (and the participants with DS) relative to participants with ASD despite significantly lower IQ found in those groups. 

We also found that the participants with DS fell between, and did not differ significantly from, the groups with FXS and ASD in terms of Phrase Repetition and Topic Repetition. Thus, these are areas of clinical concern for individuals with DS as well. Repetitive behaviors in DS have been noted in previous work [56].

The second question we addressed was whether repetitive language measured using ELS conversation correlated with other participant characteristics; namely, autistic symptomatology (both RRB and SA), CA, nonverbal intelligence and expressive language ability. Our hypothesis was only partly supported as the only significant correlations we found were for the group with ASD. These were found between all three measures of Phrase repetition and NVIQ. These significant negative correlations suggest that in individuals with ASD, repetitive language is reflective, in part, of level of cognitive ability. When the ASD group was divided into ASD-O and ASD+ID subgroups, the correlations were no longer significant, but more importantly the correlation coefficients in the ASD-ID were consistently larger than for the ASD-O group for Phrase Repetition measures and NVIQ and CA. This may indicate that in those autistic individuals with a comorbid diagnosis of ID, it is the combination of above diagnostic threshold ASD symptoms and lower IQ that results in high rates of phrase repetition. In contrast, the lack of correlation of repetition with autistic symptomatology, NVIQ, or expressive language in FXS–a disorder noted for its repetitive language—suggests that the repetitive language may be an inherent part of the FXS phenotype. 

The last question we addressed was whether it would be possible to establish initial evidence of construct validity in the ELS repetition measures by comparing it to another measure of repetitive language and an unrelated measure. After correction for multiple comparisons, no significant correlations remained between ELS-derived repetition measures and the repetitive language items from the ABC for any of the individual diagnostic groups. However, when the participants were combined into a single group, there were significant correlations between the ABC combined items and the Phrase Repetition measures and Conversational Device measures. These were the two areas targeted by the ABC questions, whereas Topic Repetition is not addressed in the ABC. The discrepancy between the results in the individual groups and the larger group could stem from several sources. Based on extensive clinical experience, it is apparent that there are high levels of variability in how caregivers perceive different behavior. For example, what one caregiver rates as a “severe problem” may be rated as “not a problem” by another caregiver. To overcome this variability, the collection of a larger sample size as well as more intentional caregiver training regarding how to complete the ABC would be useful. Nonetheless, the present findings provide some support for convergent construct validity of the phrase and CD repetition measures.

To determine discriminant validity, the ELS-derived repetition measures were compared to another subscale from the ABC that does not purport to assess repetitive language, Irritability. Prior to correction for multiple comparisons, the group with DS had one significant correlation between the total number of conversational devices used and the level of irritability as rated by a caregiver. However, once the groups were combined there were no longer significant correlations. These results provide support for the discriminant validity of the repetition measures.

Overall, the present findings replicated previous findings of increased rates of repetitive language in FXS as compared to ASD in semi-structured language samples [29,52]. The lack of correlation in the FXS group between repetition and autistic symptomatology is similar to the lack of difference between participants with FXS-O and FXS+ASD in Klusek et al. [8], although it differs from the results of Martin et al. [9,10]. The strong negative correlation between NVIQ and Phrase Repetition in the ASD group has also been mentioned in previous research, with lower NVIQ being strongly linked to increased restricted and repetitive behaviors [67]. 

### Limitations

A number of limitations of this study should be considered. First, despite being well-matched on chronological age, the sample size of the individual groups was relatively small. Additional analyses with a larger group would allow for stronger conclusions to be made and would increase the likelihood of significant correlations emerging within the individual groups. The larger sample would also allow other covariates and their interactions to be assessed, e.g., age and gender. The addition of an ASD group matched on IQ would also assist in further determining the role of cognitive ability in repetitive language. Second, the samples were drawn using only one language context, conversation. As language can vary significantly based on setting, it would be beneficial to extend this analysis to other contexts such as narratives. Third, we have not established whether this measure will be useful in treatment studies. To do so will require examination of psychometric possibilities such as practice effect, test-retest reliability, and sensitivity to change. 

## 5. Conclusions and Clinical Implications

The present study aimed to establish the utility of a repetitive language coding system in distinguishing between groups with neurodevelopmental disorders and its initial construct validity. Our results suggest that pairing the coding system described above with an ELS protocol allows for a more standardized approach to analyzing naturalistic language samples, which in turn was able to distinguish between participants with ASD and participants with FXS on multiple types of repetitive language, and between ASD, FXS and DS on one form of repetitive language. Thus, ELS procedures and the repetitive language coding scheme described here help to fill the pressing need for valid outcome measures for use in clinical trials, it can be used for individuals with wide-ranging communicative abilities. By extending its utility to the assessment of pragmatic language traits, we provide a more comprehensive understanding of language ability.

The findings that the group with FXS was significantly different than the group with ASD, with higher levels of all forms of repetition except for Conversational Devices is extremely important clinically. Given the relative lack of information regarding specific treatment approaches for communication in FXS, the majority of interventions are borrowed from the literature on autism. Given the surface similarities of these two phenotypes, this is an understandable approach. However, given that repetitive language in FXS appears to be a core part of the phenotype, and not highly linked to language or cognitive ability, it may need a separate approach in intervention. Its trend toward significance with age and ADOS-RRB could indicate that repetitive language decreases with development, as research has shown that after peaking in adolescence, RRB decrease with age in adults with FXS [68]. The finding that repetitive language in DS often fell between the levels found in FXS and ASD is also of clinical utility. The classic view of DS is that pragmatic language is a strength. However, this study adds to the growing recognition that there are specific areas of deficit in pragmatic language in that disorder. As clinicians, it’s important to view the communication profile as a whole in order to ensure that we are supporting all areas of need. 

## Figures and Tables

**Table 1:** Participant Characteristics.

	FXS (*n* = 29, 25 Male)		ASD (*n* = 30, 22 Male)		DS (*n* = 34, 18 Male)		H **
Measure	M (SD)	Range	M (SD)	Range	M (SD)	Range	
Chron Age	13.2 (4.0)	6.6–21.7	12.9 (4.7)	6.2–23.7	14.1 (5.3)	7–23.3	0.7
FSIQ ^1^	42.4 (13.1)	19.1–66.0	79.5 (17.2)	39.9–103.6	42.4 (10.9)	24.0–60.0	43.3 **
VIQ ^1^	42.9 (13.6)	19.1–65.0	77.5 (18.7)	36.1–105.9	36.9 (11.7)	7.4–53.6	45.5 **
NVIQ ^1^	44.4 (13.6)	18.3–71.4 *	82.0 (16.4)	40.5–105.7	47.6 (16.5)	20–68	45.9 **
Exp Lg ^2^	8.6 (2.9)	1–16	10.7 (3.8)	1–17	9.4 (2.9)	1–15	8.01 **
Autistic Symp^.3^	5.8 (2.4)	1–10	6.7 (1.6)	4–10	3.6 (2.5)	1–9	21.9 **

^1^ Measured using the Stanford-Binet, Fifth Edition (SB-5; [58]) using z-derived scores to avoid floor effects (Sansone, et al. [59]). ^2^ Measured using v-scores (mean of 15, s.d. of 3) from the Expressive Communication subscale of the Vineland Adaptive Behavior Scale-Second Edition (VABS-II; [60]). ^3^ Measured using the Comparison Score from the Autism Diagnostic Observation Schedule-Second Edition (ADOS-2; [61]) with higher scores indicating increased autistic symptomatology. * One participant did receive a score higher than 70 for NVIQ, but both VIQ and FSIQ were below 70 for that same participant and they were kept in analyses. ** Kruskal Wallis test statistic with ** indicating significant difference between groups at *p* < 0.05.

**Table 2:** Repetition Definitions and Examples.

Repetition Type	Definition	Example E: Examiner P: Participant	Specific Variables
*Phrase Repetition*	Phrases, dependent clauses, and/or entire C-units said more than once immediately after the first time they were uttered	P: “She was a statue” P: “She was a statue because she was a statue”	Phrase Total: Total number of C-Units containing a phrase repetition Phrase Average: Average number of repetitions of a specific variable Phrase Max: Maximum number of repetitions of a specific phrase
*Topic Repetition*	Excessive repetition of previously established topic details	E: “Who is your favorite singer?” P: “I’m gonna graduate this year”	Total number of C-Units involving a specific topic that either (1) added no new information; (2) was non-contingent to the previous statement; (3) interrupted the examiner
*Conversational Device (CD) Repetition*	Rote sayings/phrases that help direct the interaction but do not add content to the topic	P: “It’s what really inspires you to sing, *you know*.” P: “It’s a good thing that just, *you know*.” P: “*You know* what i mean.” P: “*You know*, it’s ok.”	CD Total: Total number of C-units containing a CD CD Max: Maximum number C-units containing a specific CD

**Table 3:** Median regressions for repetition measures after controlling for number of C-units.

Repetition Measure	FXS	ASD	DS		
	Median	Median	Median	*R* ^2^	*p*
Phrase Total	2	0	0	13.6043	0.0011 *
Phrase Avg	2	0	0	27.2087	<0.0001 **
Phrase Max	2	0	0	27.2087	<0.0001 **
Topic Repetition	9.0652	3.8071	6.5491	6.0121	0.0495 *
CD Total	5.721	8.4589	6.488	1.7276	0.4216
CD Max	0.5845	6.1598	1.132	46.2141	<0.0001 **

* significant at *p* < 0.05 prior to FDR correction, ** significant at *p* < 0.01 prior to FDR correction, Note: underlined cells indicate those differences that remained significant after FDR correction.

**Table 4:** Correlations between repetition measures and autistic symptomatology controlling for number of C-units.

	FXS		ASD		DS	
	ADOS RRB	ADOS SA	ADOS RRB	ADOS SA	ADOS RRB	ADOS SA
Phrase Total	0.402 *	0.109	−0.095	0.221	0.074	−0.110
Phrase Mean	0.390	0.111	−0.073	0.213	0.059	−0.127
Phrase Max	0.365	0.121	−0.073	0.213	0.018	−0.122
Topic Repetition	0.419 *	−0.118	−0.280	0.319	0.079	−0.161
CD Total	−0.450 *	−0.091	−0.381 *	−0.066	0.158	0.388 *
CD Max	−0.447 *	−0.129	−0.228	−0.017	0.048	0.250

* Significant at *p* < 0.05 prior to FDR correction.

**Table 5:** Correlations between repetition measures and NVIQ, expressive language and chronological age controlling for number of C-units.

	FXS			ASD			DS		
	CA	NVIQ	Exp Lg	CA	NVIQ	Exp Lg	CA	NVIQ	Exp Lg
Phrase Total	−0.383 *	−0.423 *	0.352	−0.276	−0.504 **	−0.053	−0.328	−0.424 *	−0.055
Phrase Mean	−0.320	−0.380	0.199	−0.278	−0.503 **	−0.059	−0.341	−0.489 *	−0.015
Phrase Max	−0.327	−0.368	0.236	−0.278	−0.503 **	−0.059	−0.348	−0.453 *	0.010
Topic Repetition	−0.373	−0.427 *	0.241	−0.389 *	−0.289	0.124	−0.450 *	−0.178	0.363 *
CD Total	0.140	0.213	−0.061	−0.254	−0.067	0.158	0.475 **	−0.161	−0.186
CD Max	0.121	0.128	−0.054	−0.252	−0.071	0.102	0.376 *	0.114	−0.083

* Significant at *p* < 0.05 prior to FDR correction, ** significant at *p* < 0.01 prior to FDR correction, Note: underlined cells indicate those correlations that remained significant after FDR correction.

**Table 6:** Correlations between repetitive language measures and ADOS-RRB, ADOS-SA, CA, and NVIQ after separating groups into ASD-O and ASD+ID and controlling for number of C-units.

	ASD-O					ASD+ID				
	ADOS RRB	ADOS SA	CA	NVIQ	Exp Lg	ADOS RRB	ADOS SA	CA	NVIQ	Exp Lg
Phrase Total	0.229	0.253	0.046	−0.288	−0.212	−0.627	−0.010	−0.320	−0.659	−0.308
Phrase Mean	0.224	0.239	0.055	−0.272	−0.193	−0.472	−0.136	−0.371	−0.489	−0.381
Phrase Max	0.224	0.239	0.055	−0.272	−0.193	−0.543	−0.137	−0.371	−0.571	−0.381
Topic Repetition	0.060	0.359	−0.200	−0.374	−0.030	−0.124	0.437	−0.169	−0.094	0.533
CD Total	−0.427	−0.007	−0.272	−0.088	0.178	0.296	0.023	−0.338	0.000	0.092
CD Max	−0.389	0.033	−0.224	0.032	0.041	0.346	0.054	−0.362	0.016	0.057

**Table 7:** Correlations between repetitive language measures and ABC summed score while controlling for number of C-units.

	FXS	ASD	DS	Combined Group
	*Repetitive speech + Repeats a word or phrase over and over*			
Phrase Total	0.182	0.182	0.256	0.256 *
Phrase Mean	0.347	0.155	0.259	0.294 **
Phrase Max	0.302	0.155	0.255	0.278 **
Topic Repetition	0.107	0.132	−0.173	0.100
CD Total	0.325	0.370 *	0.130	0.218 *
CD Max	0.214	0.423 *	0.101	0.206 *

* Significant at the *p* < 0.05 prior to FDR correction. ** Significant at the *p* < 0.01 prior to FDR correction. Note: underlined correlation coefficients indicate those correlations that remained significant following FDR correction.

**Table 8:** Correlations between repetition measures and Irritability subscale while controlling for number of C-units.

	FXS	ASD	DS	Combined Group
	*Irritability Subscale*			
Phrase Total	0.220	0.202	0.259	0.168
Phrase Mean	0.373	0.205	0.280	0.195
Phrase Max	0.344	0.205	0.234	0.142
Topic Repetition	0.142	0.044	−0.061	−0.027
CD Total	0.153		−0.399 *	−0.002
CD Max	−0.103	0.279	−0.285	0.130

* Significant at the *p* < 0.05 prior to FDR correction.

## Data Availability

Data can be made available upon request to the corresponding author.
